# Glycemic Control with Ipragliflozin, a Novel Selective SGLT2 Inhibitor, Ameliorated Endothelial Dysfunction in Streptozotocin-Induced Diabetic Mouse

**DOI:** 10.3389/fcvm.2016.00043

**Published:** 2016-10-26

**Authors:** Hotimah Masdan Salim, Daiju Fukuda, Shusuke Yagi, Takeshi Soeki, Michio Shimabukuro, Masataka Sata

**Affiliations:** ^1^Department of Cardiovascular Medicine, Institute of Biomedical Sciences, Tokushima University Graduate School, Tokushima, Japan; ^2^Department of Cardio-Diabetes Medicine, Institute of Biomedical Sciences, Tokushima University Graduate School, Tokushima, Japan

**Keywords:** SGLT2 inhibitor, hyperglycemia, endothelial function, inflammation, oxidative stress

## Abstract

**Background:**

Endothelial dysfunction caused by increased oxidative stress is a critical initiator of macro- and micro-vascular disease development in diabetic patients. Ipragliflozin, a selective sodium-glucose cotransporter 2 (SGLT2) inhibitor, offers a novel approach for the treatment of diabetes by enhancing urinary glucose excretion. The aim of this study was to examine whether ipragliflozin attenuates endothelial dysfunction in diabetic mice.

**Methods:**

Eight-week-old male C57BL/6 mice were treated with streptozotocin (150 mg/kg) by a single intraperitoneal injection to induce diabetes mellitus. At 3 days of injection, ipragliflozin (3 mg/kg/day) was administered *via* gavage for 3 weeks. Vascular function was assessed by isometric tension recording. Human umbilical vein endothelial cells (HUVEC) were used for *in vitro* experiments. RNA and protein expression were examined by quantitative RT-PCR (qPCR) and western blot, respectively. Oxidative stress was determined by measuring urine 8-hydroxy-2′-deoxyguanosine (8-OHdG) level.

**Results:**

Ipragliflozin administration significantly reduced blood glucose level (*P* < 0.001) and attenuated the impairment of endothelial function in diabetic mice, as determined by acetylcholine-dependent vasodilation (*P* < 0.001). Ipragliflozin did not alter metabolic parameters, such as body weight and food intake. Ipragliflozin administration ameliorated impaired phosphorylation of Akt and eNOS^Ser1177^ in the abdominal aorta and reduced reactive oxygen species generation as determined by urinary excretion of 8-OHdG in diabetic mice. Furthermore, qPCR analyses demonstrated that ipragliflozin decreased the expression of inflammatory molecules [e.g., monocyte chemoattractant protein-1 (MCP-1) vascular cell adhesion molecule-1 (VCAM-1), and intercellular adhesion molecule (ICAM)-1] in the abdominal aorta (*P* < 0.05). In *in vitro* studies, incubation with methylglyoxal, one of the advanced glycation end products, significantly impaired phosphorylation of Akt and eNOS^Ser1177^ (*P* < 0.01) and increased the expression of MCP-1, VCAM-1, and ICAM-1 in HUVEC.

**Conclusion:**

Ipragliflozin improved hyperglycemia and prevented the development of endothelial dysfunction under a hyperglycemic state, at least partially by attenuation of oxidative stress.

## Introduction

Diabetes is a chronic metabolic disorder characterized by inappropriate hyperglycemia due to lack of or resistance to insulin. Reactive oxygen species (ROS) generated under a hyperglycemic state play a causal role in the vascular dysfunction observed in diabetic patients. A hyperglycemic state enhances the production of ROS through various pathways. ROS generation suppresses Akt-mediated eNOS^Ser1177^ phosphorylation, leading to impairment of endothelium-dependent NO-mediated vasorelaxation (1–7). Also, ROS induce activation of redox-sensitive transcriptional factors, including NF-κB, and subsequently promote the expression of inflammatory molecules, such as intercellular adhesion molecule (ICAM)-1 and vascular cell adhesion molecule (VCAM)-1, which stimulate endothelium–leukocyte interactions and accelerate the development of vascular inflammation (8–10). Furthermore, recent studies have also suggested that production of ROS is involved in the progression of diabetes directly (11). Therefore, when we consider treatment strategies for diabetes, the regulation of oxidative stress should be taken into account.

A new class of anti-diabetic drugs targets sodium-glucose cotransporter 2 (SGLT2), the main glucose transporter in the kidney, which is located in the S1 and S2 segments of the proximal tubule and is responsible for reabsorption of >90% of glucose in primary urine (12). In recent years, SGLT2 inhibitors, which can inhibit reabsorption of filtered glucose by blocking SGLT2, have been developed and proposed as novel hypoglycemic agents for treating patients with diabetes mellitus (13). In addition, in contrast to classical anti-diabetic therapies, SGLT2 inhibitors remove glucose from the body and are thereby expected to be highly efficient in preventing oxidative stress-associated organ damage. Ipragliflozin is a novel inhibitor of SGLT2, which is used for the treatment of type 2 diabetes (14–16). However, few studies have examined the vasoprotective effects of ipragliflozin. The purpose of this study was to investigate the hypothesis that inhibition of SGLT2 by ipragliflozin ameliorates endothelial dysfunction and vascular inflammation by inhibiting oxidative stress in STZ-induced diabetic mice.

## Materials and Methods

### Animals and Drug Administration

Wild-type (C57BL/6J background) mice were purchased from Japan SLC, Inc. All experimental procedures conformed to the guidelines for animal experimentation of Tokushima University. To examine the effect of ipragliflozin on a diabetic mouse model, 8-week-old male wild-type mice were treated with 150 mg/kg streptozotocin (STZ) or vehicle (citrate buffer) *via* intraperitoneal injection one time. Adverse effects of STZ injection (e.g., weight loss, respiratory distress, and poor body condition) were reported (17). In this experiment, two mice found dead within 1 week after STZ injection. One mouse was excluded due to weight loss by more than 20% from the baseline (18). From 3 days after injection, 3 mg/kg/day ipragliflozin was administered *via* oral gavage for 3 weeks. Ipragliflozin was suspended in 0.5% carboxymethyl cellulose (CMC) solution. STZ was purchased from Sigma-Aldrich. Ipragliflozin was provided by Astellas Pharma, Inc. (Tokyo, Japan). The control group received an equal volume of CMC. Mice were maintained under a 12-h light/dark cycle with free access to normal chow and water.

### Laboratory Data

At the time of sacrifice, blood was collected from the heart into EDTA-containing tubes, and plasma was stored at −80°C until required. Plasma total cholesterol, high-density lipoprotein (HDL) cholesterol, and triglyceride levels were measured at LSI Medience Corporation (Japan). Urinary 8-hydroxy-2′-deoxyguanosine (8-OHdG) concentration in a 16-h urine collection was determined using a commercially available kit (Japan Institute for the Control of Aging, Nikken SEIL Co., Ltd.) and corrected by creatinine.

### Vascular Reactivity Assay

Analysis of vascular reactivity was performed, as described previously (19, 20). In brief, the descending thoracic aortas from mice were cut into 2-mm rings with special care to preserve the endothelium and mounted in organ baths filled with modified Krebs–Henseleit buffer (KHB; 118.4 mM NaCl, 4.7 mM KCl, 2.5 mM CaCl_2_, 1.2 mM KH_2_PO_4_, 1.2 mM MgSO_4_, 25 mM NaHCO_3_, and 11.1 mM glucose) aerated with 95% O_2_ and 5% CO_2_ at 37°C. The preparations were attached to a force transducer, and isometric tension was recorded on a polygraph. Vessel rings were primed with 31.4 mM KCl, and then pre-contracted with phenylephrine, producing submaximal (60% of maximum) contraction. After the plateau was attained, the rings were exposed to increasing concentrations of acetylcholine (Ach, 10^−9^ to 10^−4^M) and sodium nitroprusside (SNP; 10^−9^ to 10^−4^M) to obtain cumulative concentration–response curves.

### Cell Culture

Human umbilical vein endothelial cells (HUVEC) were purchased from Life Technologies and cultured in EGM-2 (Lonza). HUVEC (passage 4–6) were treated with methylglyoxal (MGO) (Sigma-Aldrich) in EBM-2 (Lonza) containing 2% FBS.

### Western Blot Analysis

Cell lysates were prepared using RIPA buffer (Wako Pure Chemical Industries, Ltd.) containing a protease inhibitor cocktail (Takara Bio Inc.) and phosphatase inhibitors (Roche). Proteins were separated by SDS-PAGE and transferred onto polyvinylidene difluoride membranes (Hybond-P; GE Healthcare). After blocking with 5% bovine serum albumin, the membranes were incubated with primary antibody against either phosphorylated-eNOS^Ser1177^, Akt, phosphorylated-Akt^Ser473^ (Cell Signaling Technology), eNOS (BD Biosciences), or β-actin (Sigma) overnight at 4°C. Horseradish peroxidase-conjugated anti-mouse Ig (Cell Signaling Technology) or anti-rabbit Ig (Chemicon) antibody was then used as the secondary antibody. Antibody distribution was visualized with ECL-plus reagent (GE Healthcare) using a luminescent image analyzer (LAS-1000, Fuji Film).

### Real-time Polymerase Chain Reaction

Total RNA was extracted from the aorta and cells using an illustra RNAspin RNA Isolation Kit (GE Healthcare). cDNA was synthesized from 100 ng of total RNA using a QuantiTect Reverse Transcription kit (Qiagen). Quantitative RT-PCR (qPCR) was performed on an Mx3000P (Agilent Technologies) using Power SYBR Green PCR Master Mix (Applied Biosystems). Mouse PCR primers were as follows: VCAM-1, sense 5′-CCCGTCATTGAGGATATTGG-3′ and antisense 5′-GGTCATTGTCACAGCACCAC-3′; ICAM-1, sense 5′-TTCACACTGAATGCCAGCTC-3′ and antisense 5′-GTCTGCTGAGACCCCTCTTG-3′; monocyte chemoattractant protein (MCP)-1, sense 5′-CCACTCACCTGCTGCTACTCAT-3′ and antisense 5′-TGGTGATCCTCTTGTAGCTCTCC-3′; F4/80, sense 5′-TGCATCTAGCAATGGACAGC-3′ and antisense 5′-GCCTTCTGGATCCATTTGAA-3′; and β-actin, sense 5′-CCTGAGCGCAAGTACTCTGTGT-3′ and antisense 5′-GCTGATCCACATCTGCTGGAA-3′. Human PCR primers were as follows: VCAM-1, sense 5′-ATGAATTCGAACCCAAACA-3′ and antisense 5′-CCTGGCTCAAGCATTGTCATA-3′; MCP-1, sense 5′-CCCCAGTCAACCGCTGTTAT-3′ and antisense 5′-AGATCTCCTTGGCCACAATG-3′; ICAM-1, sense 5′-TGATGGGCAGTCAACAGCTA-3′ and antisense 5′-GGGTAAGGTTCTTGCCCACT-3′; and glyceraldehyde 3-phosphate dehydrogenase (GAPDH), sense 5′-TGGGTGTGAACCATGAGAAG-3′ and antisense 5′-GCTAAGCAGTTGGTGGTGC-3′. Data are expressed in arbitrary units that were normalized by β-actin or GAPDH.

### Statistical Analysis

All results are expressed as mean ± SEM. Comparison of parameters between two groups was performed with unpaired Student’s *t*-test. Differences between multiple groups were analyzed by one-way analysis of variance (ANOVA), followed by Tukey’s *post hoc* analysis. Comparisons of dose–response curves were made by two-factor repeated-measures ANOVA, followed by Tukey’s *post hoc* test for comparison between groups. A value of *P* < 0.05 was considered significant.

## Results

### Ipragliflozin Decreased Blood Glucose Level in STZ-Induced Diabetic Mice

**Figure 1:**
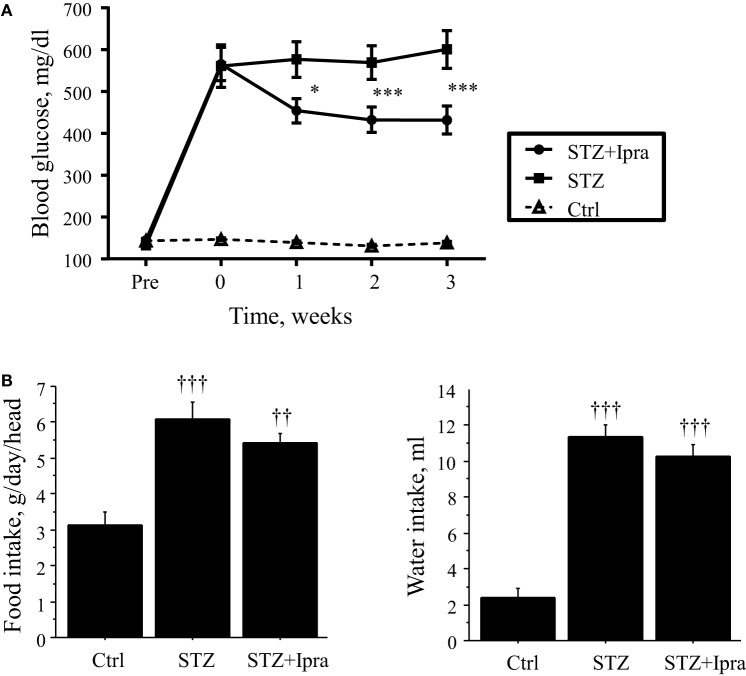
**Effect of ipragliflozin administration on non-fasting blood glucose level**. **(A)** Ipragliflozin administration to STZ-induced diabetic mice significantly decreased non-fasting blood glucose level compared with vehicle. **(B)** STZ injection significantly increased food and water intake, although there was no difference between the ipragliflozin-treated and vehicle-treated groups. Ctrl, control (non-STZ injected); Ipra, ipragliflozin. *P < 0.05 and ***P < 0.001 vs. STZ group. ^††^*P* < 0.01 and ^†††^*P* < 0.001 vs. Ctrl group. Data represent mean ± SEM.

In this study, STZ injection markedly elevated the non-fasting blood glucose level compared with that in control mice. Ipragliflozin significantly decreased the non-fasting blood glucose level compared with the vehicle-treated group (Figure 1A). There were no significant differences in the plasma levels of total cholesterol, HDL cholesterol, and triglyceride between the ipragliflozin-treated and vehicle-treated groups in STZ-induced diabetic mice (Table 1). STZ injection significantly reduced body weight and increased food and water intake, although there was no difference between the ipragliflozin-treated and vehicle-treated groups, as shown in Table 1 and Figure 1B. Ipragliflozin increased urine volume (vehicle vs. ipragliflozin; 9.5 ± 1.1 vs. 11.2 ± 2.0 ml/day) and urinary glucose excretion (vehicle vs. ipragliflozin; 617.7 ± 60.5 vs. 802.7 ± 133.5 mg/day) in diabetic mice, although they did not reach statistical significance.

**Table 1:** **Effects of ipragliflozin treatment on metabolic parameters**.

	Ctrl (*N* = 11)	STZ (*N* = 13)	STZ + Ipra (*N* = 14)
Blood glucose, mg/dl	138.0 ± 3.8	600.6 ± 45.0^†††^	431.8 ± 33.6***^†††^
Body weight, g	25.3 ± 0.4	20.4 ± 0.7^†††^	20.2 ± 0.6^†††^
Total cholesterol, mg/dl	86.0 ± 5.9	113.8 ± 12.0	130.1 ± 13.0^†^
Triglyceride, mg/dl	66.5 ± 9.8	137.9 ± 32.6	157.3 ± 37.0^†^
HDL-cholesterol, mg/dl	49.5 ± 3.5	62.0 ± 6.4	71.0 ± 6.3

*Ctrl; control, STZ; streptozotocin, Ipra; ipragliflozin; HDL; high-density lipoprotein*.

*All values are mean ± SEM*.

^†^P < 0.05 and ^†††^P < 0.001 vs. control and ***P < 0.001 vs. STZ.

### Ipragliflozin Ameliorated Endothelial Dysfunction in STZ-Induced Diabetic Mice

**Figure 2:**
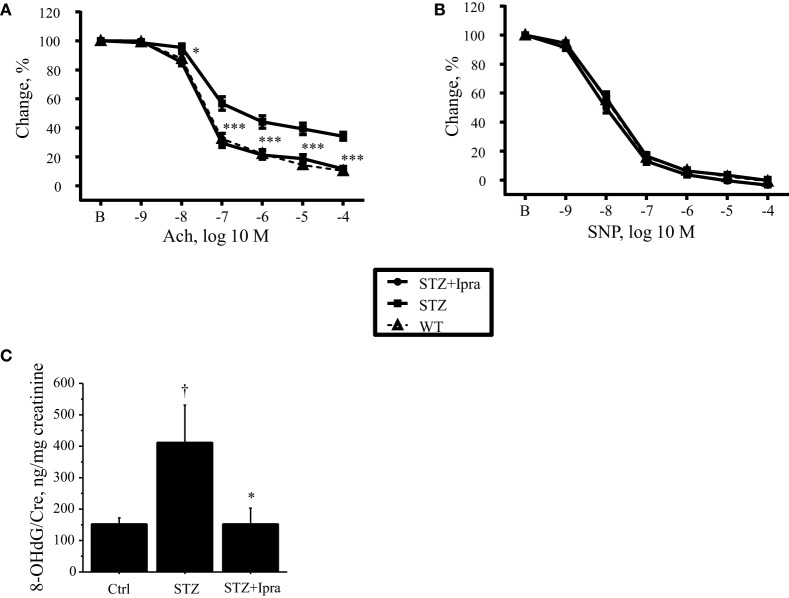
**Ipragliflozin attenuated endothelial dysfunction in STZ-induced diabetic mice**. **(A,B)** Vascular reactivity to Ach or SNP was determined using aortic rings isolated from ipragliflozin- or vehicle-administered STZ-induced diabetic mice and control mice. Ipragliflozin administration for 3 weeks ameliorated endothelium-dependent vasodilation in response to Ach compared with the non-treated diabetic group **(A)**. Vasorelaxation in response to SNP did not differ among the three groups **(B)**. **(C)** STZ-induced diabetic mice had significantly higher urine 8-OHdG level compared with non-diabetic control mice. Ipragliflozin administration significantly ameliorated it compared with vehicle treatment. *n* = 10–12 per group. Ctrl, control (non-STZ injected); Ipra, ipragliflozin. *P < 0.05 and ***P < 0.001 vs. STZ group. ^†^*P* < 0.05 vs. Ctrl group.

In diabetes, vascular dysfunction is characterized by impaired endothelial function due to increased oxidative stress. Therefore, to investigate the effects of ipragliflozin on endothelial function, vascular response was examined. Endothelium-dependent vasodilation in response to Ach was impaired in STZ-induced diabetic mice compared with that in the control group (*P* < 0.001); however, ipragliflozin administration significantly ameliorated the impairment of endothelium-dependent vasodilation (*P* < 0.001) (Figure 2A). On the other hand, endothelium-independent relaxation in response to SNP did not differ between the ipragliflozin-treated group and vehicle-treated group (Figure 2B). Compared with non-diabetic control, STZ injection increased urinary excretion of 8-OHdG (*P* < 0.05), which was significantly ameliorated by ipragliflozin administration (*P* < 0.05) (Figure 2C).

### Effects of Ipragliflozin on Endothelial Function in STZ-Induced Diabetic Mice

**Figure 3:**
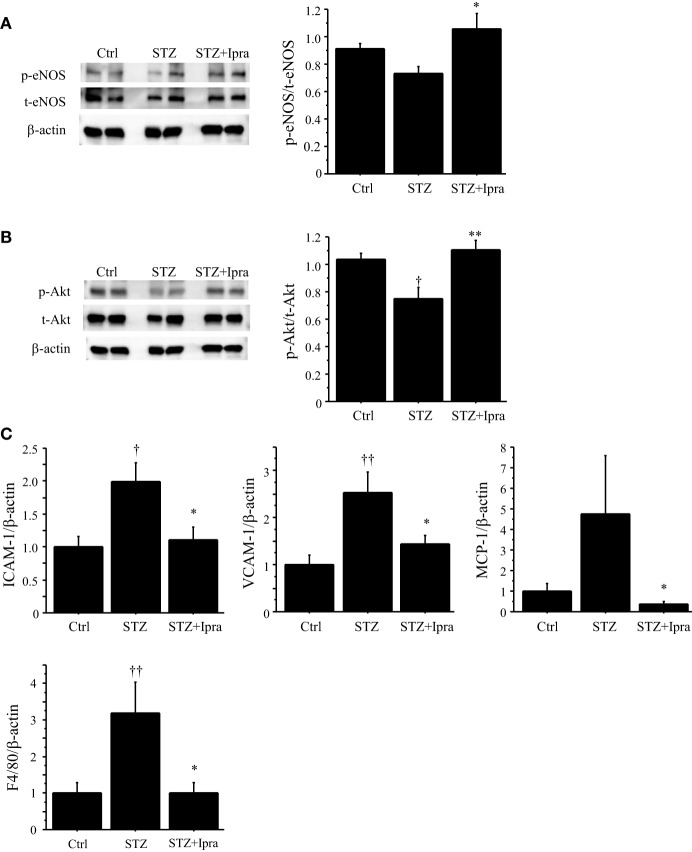
**Ipragliflozin ameliorated vascular dysfunction induced by hyperglycemic state**. **(A,B)** Treatment with ipragliflozin improved phosphorylation of eNOS at Serine 1177 **(A)** and Akt **(B)** in the abdominal aorta compared with the non-treated group. **(C)** Results of qPCR demonstrated that ipragliflozin administration reduced ICAM-1, VCAM-1, MCP-1, and F4/80 expression in the abdominal aorta of STZ-induced diabetic mice. *n* = 8 per group. Ctrl, control (non-STZ injected); Ipra, ipragliflozin. *P < 0.05 and **P < 0.01 vs. STZ group. ^†^*P* < 0.05 and ^††^*P* < 0.01 vs. Ctrl group. All values are mean ± SEM.

To investigate the mechanism by which ipragliflozin improved endothelial function, we examined phosphorylation of eNOS and Akt. Phosphorylation of eNOS at Ser1177, which increases eNOS activity, was impaired in STZ-induced diabetic mice; however, ipragliflozin ameliorated this impairment (*P* < 0.05) (Figure 3A). STZ-induced diabetic mice also showed reduction of Akt phosphorylation, and this downregulation of Akt phosphorylation was restored by ipragliflozin treatment (*P* < 0.01) (Figure 3B).

We also examined the effects of ipragliflozin on the expression of inflammatory molecules in the abdominal aorta using qPCR. Ipragliflozin treatment decreased the expression of ICAM-1, VCAM-1, and MCP-1 (*P* < 0.05) in the abdominal aorta compared with vehicle treatment in STZ-induced diabetic mice (Figure 3C), suggesting inhibition of inflammatory activation of endothelial cells by ipragliflozin in a diabetic condition. In fact, the expression of F4/80, a macrophage marker, in the aorta was reduced in ipragliflozin-treated diabetic mice (Figure 3C).

### Effects of Glucose Toxicity on Endothelial Cell Function

**Figure 4:**
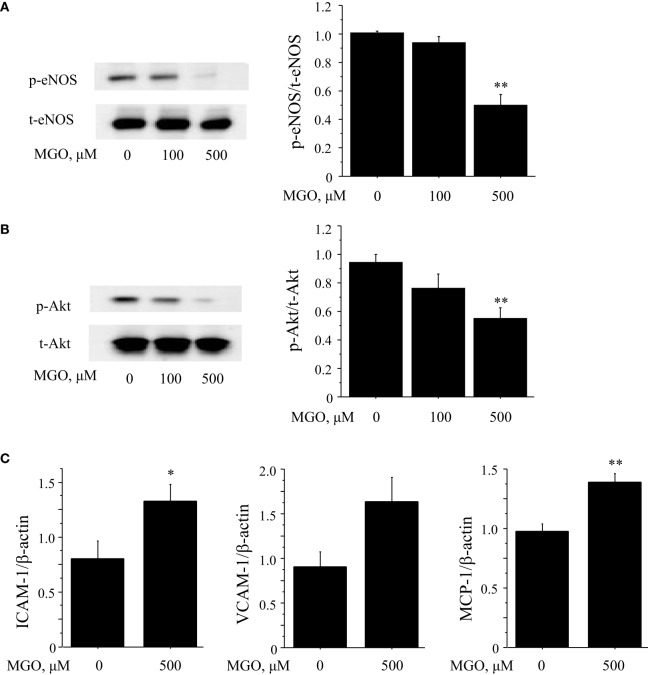
**Glucose toxicity induced endothelial cell dysfunction**. **(A,B)** To investigate the effects of glucose toxicity on endothelial function, HUVEC were treated with MGO, a precursor of AGE. Incubation with MGO for 4 h reduced the phosphorylation at Ser1177 **(A)** and Akt **(B)** in HUVEC. **(C)** Results of qPCR demonstrated that incubation of HUVEC with 500 μM MGO for 4 h significantly increased the expression of ICAM-1 and MCP-1 and tended to increase VCAM-1 (*P* = 0.06). *n* = 4 per group. Ctrl, control; MGO, methylglyoxal. *P < 0.05 and **P < 0.01 vs. Ctrl. All values are mean ± SEM.

Hyperglycemia promotes oxidative stress by contributing to the production of advanced glycation end products (AGEs), leading to an increase in intracellular oxidants, a decrease in bioavailability of endothelium-derived NO, and the development of vascular inflammation. Therefore, to investigate the effects of AGE on endothelial cell function, *in vitro* studies using HUVEC were performed. Incubation with MGO, a major precursor of AGEs, significantly reduced the phosphorylation of eNOS at Ser1177 and Akt (*P* < 0.01) (Figures 4A,B). MGO also increased the expression of ICAM-1, VCAM-1, and MCP-1 in HUVEC (Figure 4C). These results correspond to the results we observed in *in vivo* studies, suggesting that ipragliflozin treatment attenuated endothelial dysfunction by reducing glucose toxicity.

## Discussion

SGLT2 inhibitors excrete excess glucose into the urine and exhibit an anti-hyperglycemic effect (18, 21, 22). Hyperglycemia has been recognized as a primary factor in endothelial dysfunction, leading to the development of diabetic micro- and macro-vascular complications in diabetic patients. In this study, we demonstrated that administration of ipragliflozin, a SGLT2 inhibitor, to STZ-induced diabetic mice lowered the blood glucose level and prevented the development of endothelial dysfunction and vascular inflammation, at least partially through a reduction of oxidative stress. Accumulating evidence suggests cardioprotective effects of SGLT2 inhibitors (23, 24). Our study might provide one of the mechanisms for these effects.

Previous studies have revealed that the cardiovascular consequences of diabetes mellitus are associated with oxidative stress (25–27). A hyperglycemic state increased non-enzymatic glycosylation of proteins and subsequent formation of AGE, which interact with the receptor for AGE (RAGE) on the plasma membrane and promote the production of ROS, contributing to vascular complications (28). Also, hyperglycemic state can activate polyol pathways and PKC, promoting ROS generation (29, 30). Previous basic and clinical studies have shown that ROS generation suppresses Akt-mediated eNOS^Ser1177^ phosphorylation, leading to the impairment of endothelium-dependent NO-mediated vasorelaxation (1–7). In this study, ipragliflozin improved endothelium-dependent vasodilation in STZ-induced diabetic mice, indicating its protective effects on endothelial cell function in a hyperglycemic state. Our results demonstrated that ipragliflozin increased the phosphorylation of eNOS at Ser1177 and Akt in the aorta of diabetic mice. Therefore, the improvement of vascular endothelial function by ipragliflozin in diabetic mice seems to be at least partially attributable to the improvement of eNOS function in a hyperglycemic state. The results of *in vitro* experiments demonstrating attenuation of Akt and eNOS phosphorylation in HUVEC treated with MGO, the precursor of AGE, support our *in vivo* results. These results suggest that a reduction of oxidative stress through the excretion of excess glucose is one of the mechanisms of the cardioprotective effects of SGLT2 inhibitors. In addition, our present study demonstrated that the glucose-lowering effect of ipragliflozin in STZ-induced diabetic mice significantly decreased oxidative stress as determined by urinary excretion of 8-OHdG. Previous studies have also shown that treatment with empagliflozin, another SGLT2 inhibitor, for 7 or 10 weeks improved endothelial function in STZ-induced and obesity-induced diabetic animal models (22, 31), whereas, in this study, we observed improvement of endothelial function in an even shorter treatment period, suggesting the reliability and effectiveness of glucose-lowering therapy with SGLT2 inhibitors for improving endothelial function.

A hyperglycemic state also induces proinflammatory activation of endothelial cells. Several studies performed in diabetic patients, animals, and high-glucose-treated endothelial cells demonstrated that hyperglycemia-induced ROS formation in various cells (32–35) induces the activation of NF-κB and subsequent inflammation (8–10). Our present study showed that hyperglycemia induced by STZ increased the expression of inflammatory molecules, such as ICAM-1 and VCAM-1, in the aorta, which was suppressed by ipragliflozin treatment. The results of *in vitro* experiments in which MGO increased the expression of these inflammatory molecules in HUVEC support our *in vivo* results. These results also suggest cardioprotective effects of ipragliflozin.

This study was performed in a STZ-induced diabetic model, a model of type 1 diabetes. It is unclear whether the present results from a type 1 diabetes model are translatable to type 2 diabetes, which is the most common type of diabetes in humans. This is a major limitation for translating our findings to the clinical situation. However, several studies have already demonstrated that other SGLT2 inhibitors prevented vascular dysfunction in type 2 diabetes (13, 15, 21, 31). Also, several studies reported the potential of SGLT2 inhibitors, such as dapagliflozin, in type 1 diabetes (36). Therefore, the results of this study support the concept that the glucose-lowering effect of SGLT2 inhibitors is associated with cardioprotective effects of this class of anti-diabetic drug. In this experiment, urine volume and urinary glucose excretion between vehicle-treated and ipragliflozin-treated diabetic mice did not differ significantly. SGLT2 inhibitors increase urinary glucose excretion and consequently increase urine volume. However, several studies reported that long-term treatment did not increase urinary glucose excretion and urine volume significantly due to the reduction of blood glucose level (31, 37). The results of our present study corresponded with those studies.

In conclusion, our results demonstrated that ipragliflozin improved endothelial dysfunction in STZ-induced diabetic mice. The beneficial effect of ipragliflozin was associated, at least in part, with a reduction of oxidative stress. Taken together with the results of previous studies, our results reveal one of the mechanisms of the cardioprotective effects of SGLT2 inhibitors. Further studies are needed to elucidate these mechanisms, although our present study supports the results of recent clinical studies that suggested cardioprotective effects of SGLT2 inhibitors (23, 24).

## Author Contributions

HS performed most of the experiments, interpreted the results, and prepared the manuscript. DF designed the experiments, interpreted the results, and prepared the manuscript. SY and TS were involved in discussions. MiS contributed to data interpretation and critical reading of the manuscript. MaS interpreted the data, prepared the manuscript, and supervised this study. All authors discussed the results and commented on the manuscript.

## Conflict of Interest Statement

Dr Masataka Sata received research funding and honorariums from Astellas Pharma, Inc. Other authors declare that they have no conflict of interest.

## Abbreviations

8-OHdG, 8-hydroxy-2′-deoxyguanosine; Ach, acetylcholine; CMC, carboxymethyl cellulose; GAPDH, glyceraldehyde 3-phosphate dehydrogenase; HDL, high-density lipoprotein; HUVEC, human umbilical vein endothelial cells; ICAM-1, intercellular adhesion molecule-1; MCP-1, monocyte chemoattractant protein-1; MGO, methylglyoxal; qPCR, quantitative RT-PCR; RAGE, receptor for AGE; ROS, reactive oxygen species; SGLT2, sodium-glucose cotransporter 2; SNP, sodium nitroprusside; STZ, streptozotocin; VCAM-1, vascular cell adhesion molecule-1.
